# The ‘mitochondrial guardian’ α-amyrin links colourful fruit consumption to cognitive resilience

**DOI:** 10.1080/15548627.2026.2664599

**Published:** 2026-04-29

**Authors:** Shu-Qin Cao, Juan Ignacio Jiménez-Loygorri, Patricia Boya, Evandro F. Fang

**Affiliations:** a Department of Clinical Molecular Biology, University of Oslo and Akershus University Hospital, Lørenskog, Norway; b Tumour Biology Programme, Spanish National Cancer Research Centre (CNIO), Madrid, Spain; c Departament of Neuroscience and Movement Science, Section Medicine, University of Fribourg, Fribourg, Switzerland; d The Norwegian Centre on Healthy Ageing (NO-Age) and the Norwegian National Anti-Alzheimer’s Disease (NO-AD) Networks, Oslo, Norway

**Keywords:** α-amyrin, DLK, lipid-like molecule, mitophagy, ULK1

## Abstract

Mitochondrial quality control is essential for maintaining neuronal function and resilience during aging, yet pharmacological strategies that effectively restore mitophagy to maintain mitochondrial homeostasis remain limited. Emerging evidence suggests that dietary molecules may influence mitochondrial health, although the underlying mechanisms are largely unknown. Here, we summarize our recent finding whereby we have identified a robust mitophagy inducer: α-amyrin (αA). This molecule is a lipid-like pentacyclic triterpenoid abundant in edible plants, such as passion fruit. Mechanistically, αA targets dual leucine zipper kinase (DLK), a neuron-enriched stress kinase that plays a central role in axonal degeneration signaling. Under pathological stress, DLK activates the degeneration mediator SARM1, which can sequester the key autophagy/mitophagy protein ULK1 leading to compromised autophagy (including mitophagy). By specifically binding to DLK, αA releases ULK1 from SARM1-mediated restriction and promotes ULK1-dependent mitophagy, restoring mitochondrial homeostasis. This mechanism reveals the DLK-SARM1-ULK1 cascade as a previously underappreciated regulatory interface linking neuronal stress signaling to mitochondrial surveillance pathways. More broadly, these findings introduce lipid-like dietary molecules as potential “mitochondrial guardians” that preserve organelle integrity through physiological activation of mitophagy. Targeting the DLK-SARM1-ULK1 axis with such molecules may represent a promising strategy for maintaining mitochondrial health and mitigating neurodegenerative processes associated with aging.

## Are lipid-like dietary molecules the missing pharmacological compounds for safeguarding mitochondrial health in aging brains?

Mitochondrial function is the central hub for cellular homeostasis via integrating bioenergetics, redox signaling, and metabolic adaptation. Beyond the production of adenosine triphosphate (ATP), the primary energy currency, mitochondria also orchestrate cellular stress responses, apoptosis, and innate immunity, to maintain cellular function and organismal health.

Mitochondrial dysfunction and impaired autophagy (including compromised mitophagy) are hallmarks of both biological aging and neurodegenerative diseases, yet pharmacological strategies to restore mitochondrial quality control, and especially with translational potential, remain limited. While many reported mitophagy modulators have shown promise in preclinical studies, some common limitations are poor brain penetration and high toxicity, limiting their translational potential. It is thus urgent to identify potent mitophagy inducers with improved pharmacokinetics and high translational potential.

**Figure 1.**
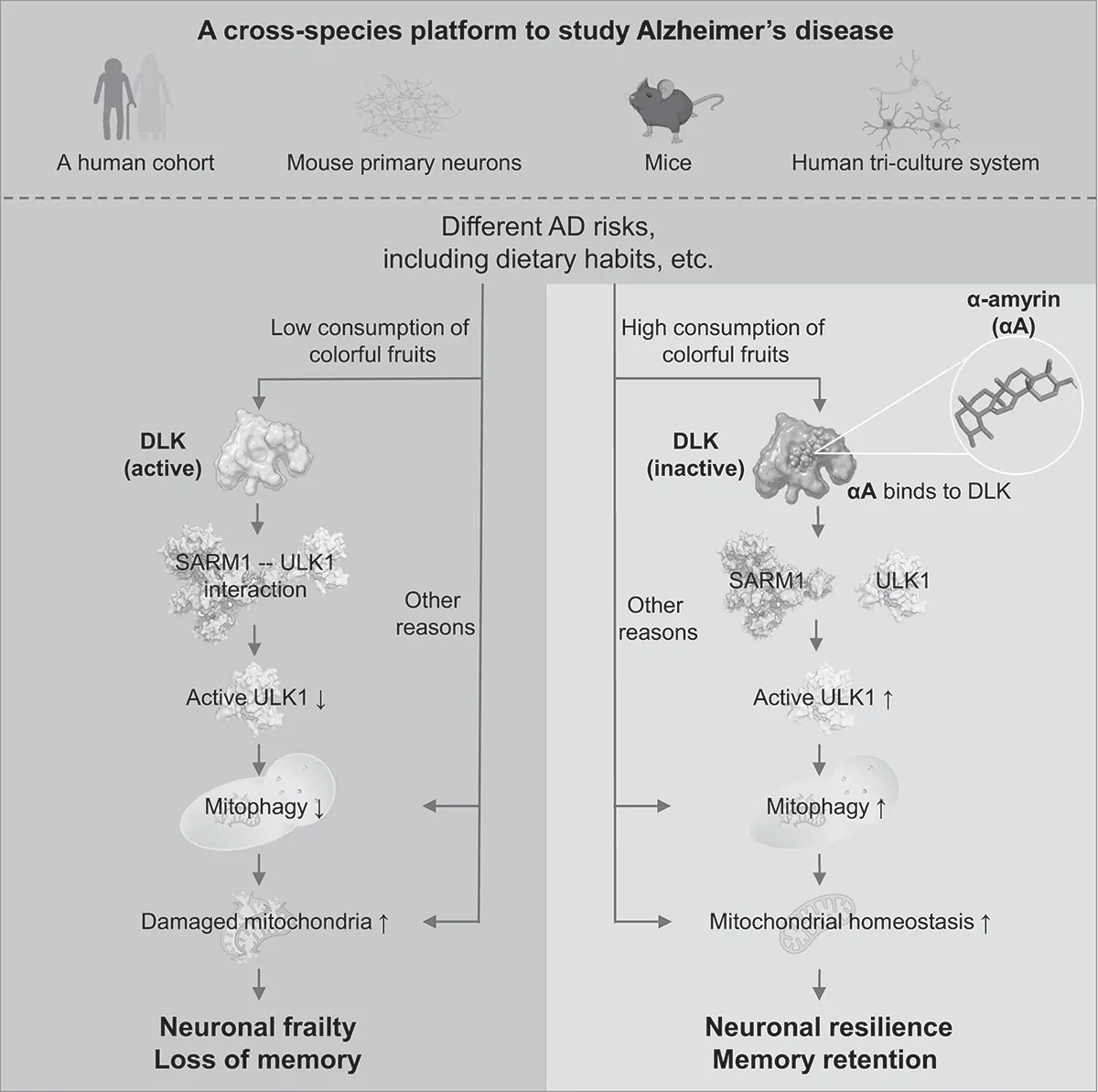

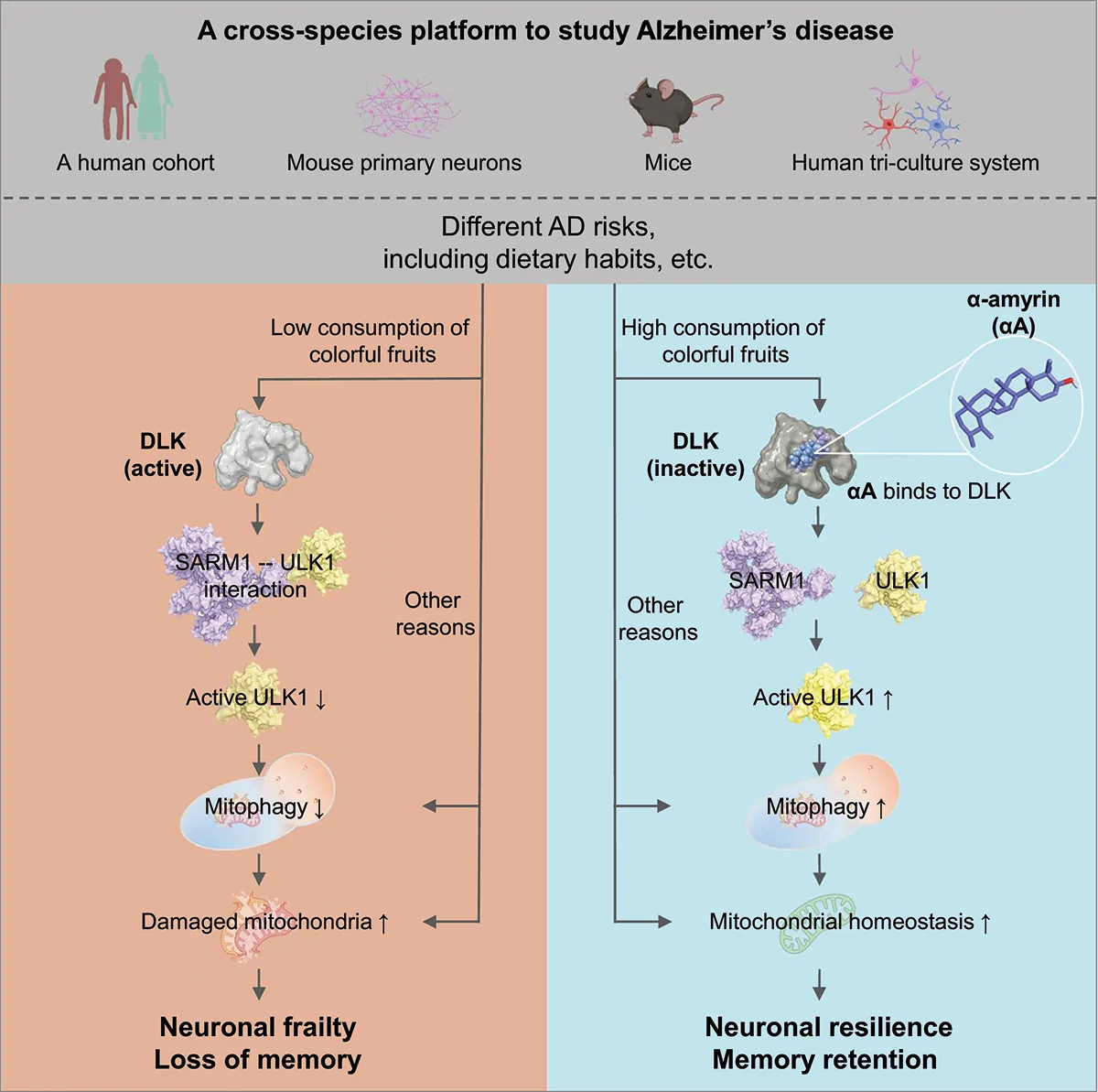
A schematic work model on how α-amyrin inhibits ad pathologies. The mitophagy inducer α-amyrin (αA) is available in colourful fruits such as passion fruit, and is able to enhance neuronal resilience and mitigate ad pathologies in a cross-species ad plateform. At the protein level, αA binds and inhibits DLK leading to the release of ULK1 from the SARM1-ULK1 complex, enabling free ULK1 to participate in autophagy and mitophagy. In different model systems of ad, restored mitophagy with enhanced mitochondrial homeostasis via modulating the DLK-SARM1-ULK1 axis, further mechanistic and clinical studies needed. For the figure, the 3D protein structures were generated using Gemini’s AI image generator with Nano Banana2, and part of the diagram were generated using BioRender.

In our recent study [1], we identify α-amyrin (αA), a pentacyclic triterpenoid abundant in plants with edible peel, as a dietary mitophagy activator with robust neuroprotective effects. αA belongs to a class of lipid-like natural products with sterol-like scaffolds and strong membrane affinity, properties that confer favorable pharmacokinetics and brain exposure. αA is affluent in passion fruit, and is also detectable in other fruits and vegetables such as Asian pear, sweet cherry, olive, and European cranberry. A summary of our study is presented (Figure 1).

## Dietary α-amyrin improves cognition and mitigates tau pathology via mitochondrial quality control

Using a longitudinal human cohort from the Shanghai Aging Study, we observed that dietary intake of fruits and vegetables correlated with reduced plasma p-Tau217 levels and lower dementia risk, highlighting diet as a modifiable factor influencing neurodegeneration. Guided by this observation, we screened dietary compounds from colorful fruit and vegetables and identified αA as a top lipid-like candidate with potent anti-Alzheimer´s disease (AD) activity.

In mouse models, αA significantly improved cognitive performance and reduced pathological Tau phosphorylation. These benefits were accompanied by reduction of damaged mitochondria, suggesting that mitochondrial quality control is a key mediator of αA’s neuroprotective effects.

## α-amyrin enhances mitochondrial function and resilience under stress

To mechanistically dissect how αA influences mitochondrial biology, we evaluated mitochondrial respiration in ARPE-19 cells, a neuroectoderm-derived cell line with exceptionally high metabolic activity and mitochondrial dependence. αA increased basal oxygen consumption, ATP production, maximal respiration, and spare respiratory capacity, indicating enhanced mitochondrial bioenergetics and stress resilience.

Beyond respiration, αA induced autophagy and mitophagy without disrupting mitochondrial membrane potential or increasing reactive oxygen species, suggesting that it promotes mitochondrial turnover without inducing mitochondrial damage. These results position αA as a mitochondrial “guardian” molecule that preserves organelle integrity rather than triggering stress-induced mitophagy.

## The DLK – SARM1–ULK1 axis as a druggable mitophagy signaling node

To elucidate the mechanism underlying αA activity, we identified dual leucine zipper kinase (DLK; also named MAP3K12) as a principal molecular target through kinome profiling and structural analyses. DLK is a neuron-enriched stress kinase that plays a pivotal role in neuronal fate determination. While transient DLK signaling contributes to axonal remodeling and regeneration, sustained activation under pathological stress drives neurodegenerative cascades partly through activation of the axon degeneration mediator SARM1. SARM1 can recruit and sequester ULK1, thereby restricting ULK1-dependent autophagy and mitophagy and linking neuronal stress signaling to impaired mitochondrial quality control. Our findings extend this framework by showing that αA modulates the DLK-SARM1-ULK1 cascade to release ULK1 and promote mitophagy, positioning DLK as a regulatory node that integrates neuronal stress responses with mitochondrial surveillance pathways.

While we do not exclude the possibility of αA to regulate autophagy-independent pathways contributing to reduced AD pathologies, our interventional experiments (KD of ULK1 and the use of a ULK1 inhibitor) support an argument that αA induced inhibition of AD pathologies was significantly dependent on ULK1.

## Implications for aging, diet, and therapeutic development

Our findings extend the concept of dietary mitophagy activators beyond caloric restriction and exercise mimetics, highlighting specific lipid-like metabolites as modulators of mitochondrial quality control. This work bridges nutritional epidemiology, chemical biology, and neurodegeneration research, suggesting that diet-derived lipid-like molecules can directly engage intracellular signaling pathways to maintain mitochondrial integrity.

The translational implications are significant. Targeting the DLK – SARM1–ULK1 mitophagy axis with lipid-like molecules could provide a new therapeutic strategy for AD and other neurodegenerative disorders. Moreover, the favorable drug-like properties of triterpenoids position them as attractive candidates for long-term interventions aimed at promoting mitochondrial health and healthy aging.

## Concluding perspective

Overall, our findings propose lipid-like dietary molecules as a previously underappreciated pharmacological space for safeguarding mitochondrial health. By identifying αA as a neuronal mitophagy activator acting through the DLK-SARM1-ULK1 axis, we establish a mechanistic link between diet, mitochondrial quality control, and neurodegeneration. These findings open new avenues for developing mitochondria-targeted therapeutics and suggest that the lipid chemical space may hold untapped potential for interventions that promote cognitive resilience and longevity.

## Data Availability

No new data were generated or analyzed in support of this publication
